# Optimal Timing of Ovulation Triggering to Achieve Highest Success Rates in Natural Cycles—An Analysis Based on Follicle Size and Oestradiol Concentration in Natural Cycle IVF

**DOI:** 10.3389/fendo.2022.855131

**Published:** 2022-05-26

**Authors:** Anja Helmer, Isotta Magaton, Odile Stalder, Petra Stute, Daniel Surbek, Michael von Wolff

**Affiliations:** ^1^ University Women’s Hospital, Division of Gynaecological Endocrinology and Reproductive Medicine, Inselspital, Bern, Switzerland; ^2^ Clinical Trial Unit (CTU) Bern, University of Bern, Bern, Switzerland; ^3^ University Women’s Hospital, Department of Obstetrics and Feto-Maternal Medicine, Inselspital, Bern, Switzerland

**Keywords:** follicle, follicle size, oestradiol, *in vitro* fertilization, natural cycle IVF, ovulation triggering, implantation rate, live birth rate

## Abstract

**Introduction:**

Timing of ovulation triggering is essential in infertility treatments including treatments based on natural menstrual cycles. However, data on follicle size and oestradiol (E2) concentration are limited. Therefore, the model of natural cycle IVF (NC-IVF) was applied to provide more detailed information on these parameters to better schedule the optimal time for triggering ovulation.

**Materials and Methods:**

A retrospective cross-sectional analysis of 606 monofollicular NC-IVF cycles was performed at a university-based IVF centre from 2016 to 2019. Follicle size and E2 and LH serum concentrations were evaluated on day -5 to 0 (day 0 = day of oocyte retrieval). Ovulation was triggered if follicle size was 14–22 mm. Patients with irregular cycles, endometriosis >II°, cycles with azoospermia or cryptozoospermia and cycles with inconsistent data were excluded. All parameters were analysed inter- and intraindividually, and associations of the parameters were evaluated. Associations were adjusted for age, cause of infertility and number of previous transfers.

**Results:**

The mean age of women undergoing NC-IVF was 35.8 ± 4.0 years. Follicle size increased by 1.04 ± 0.03 mm, and E2 concentration by 167 ± 11.0 pmol/l per day.

Based on a multivariate adjusted mixed model with follicle size, E2 and their interaction, the number of retrieved oocytes was associated with E2 concentration (aOR 1.91, 95% CI: 1.03–3.56; p = 0.040). Maturity of oocytes was associated not only with E2 concentration (aOR 2.01, 95% CI: 1.17–3.45; p = 0.011) but also with follicle size (aOR 1.27, 95% CI: 1.01–1.60; p = 0.039), as was the interaction of both parameters (aOR 0.96, 95% CI: 0.93–0.99; p = 0.017).

LH surge was calculated to start in 25% of cases at an E2 level of 637 pmol/l, in 50% of cases at 911 pmol/l and in 75% of cases at an E2 level of 1,480 pmol/l.

The live birth rate per follicle aspiration cycle was (non-significantly) higher in cycles with follicles sizes at the time of oocyte retrieval of 18–22 mm (7.7%–12.5%) versus in cycles with follicles sizes of 14–17 mm (1.6%–4.3%).

**Conclusion:**

The study contributes to an optimization of infertility treatments involving natural cycles. The study gives guidance about the number of days required after follicle monitoring to schedule the optimal time for triggering ovulation.

## Introduction

Many infertility treatments are performed in natural cycles in which only medication to trigger ovulation is administered. One reason is that low-dose monofollicular ovarian gonadotropin stimulation does not increase the pregnancy rate in regular cycles as shown for timed intercourse, intrauterine inseminations and IVF thawing cycles (1, 2).

Furthermore, natural cycle-based treatments can even be beneficial for patients. In *in vitro* fertilization (IVF), it was shown that natural cycle-based thawing cycles bear a lower risk of pregnancy-associated complications such as hypertensive disorders, preeclampsia, large for gestational age babies and macrosomia compared to artificial cycle-based thawing cycles (3). In women with a very low ovarian reserve, natural cycle IVF (NC-IVF) has been shown to be more effective than gonadotropin-stimulated IVF (4) and perinatal outcome of children born after NC-IVF seems to be better than in children born after gonadotropin-stimulated IVF (5). Furthermore, treatments based on natural cycles do not require gonadotropin injections and do not require luteal phase support (6) which can cause some discomfort, especially if applied vaginally (7).

However, natural cycles require perfect timing of the hCG trigger to achieve the highest possible success rate. A too early triggering might result in immature oocytes, immature endometrium and luteal phase insufficiency. Moreover, if the trigger is timed too late, spontaneous LH surge might already have occurred resulting in a wrong timing of intercourse and insemination or oocyte retrieval (8).

Some previous studies have already provided basic data on follicle growth and the association of oestradiol (E2) concentration and LH surge in natural cycles. The studies described an average follicle growth per day of 2.5 mm (9) or 1.42 mm (10), a mean size of the follicle at the time of ovulation of 20.2 mm (11) or 21.4 mm (12) and an average E2 level 1 day before the luteinizing hormone (LH) peak of 1087 pmol/L (12). They also analysed the relation between E2 and LH during the LH peak (13).

However, even though these studies provide substantial data on the physiology and endocrinology of folliculogenesis, they are based only on small sample sizes, have not been generated in NC-IVF cycles and are not linked with success rates.

Data on the ideal follicle size to achieve the highest success rates have already been evaluated in gonadotropin-stimulated IVF cycles (14–16), but only in one small study in NC-IVF cycles (17). As the growth dynamic of follicles is different in stimulated cycles (10), because stimulated IVF is associated with a multifollicular response and as E2 concentrations cannot be analysed per follicle in stimulated IVF, the results of these studies cannot be interpreted in the context of natural cycles.

We therefore used the model of NC-IVF to analyse a large number of patients and IVF cycles to evaluate the dynamics of follicular growth, E2 concentration during the most relevant few days before ovulation triggering and the association between E2 concentration and beginning LH surge. This time period is crucial for the clinician to estimate the onset of the LH surge and to define the best timing for ovulation triggering. The goal of the study was to provide the most realistic data and therefore also to describe the heterogeneity of different natural cycles to provide data which give the best guidance to clinicians to optimize natural cycle-based treatments.

## Methods

### Study Population and Participants

This is a retrospective, observational single-centre study performed between 2016 and 2019. 290 women, 22–42 years of age with a regular menstrual cycle (25–35 days) and basal FSH and LH concentration <10 IU/l and LH concentration before ovulation triggering <20 IU/l undergoing 606 NC-IVF cycles, were included in the study. A beginning LH surge was defined as LH concentration between 10 and <20 IU/l (18). 138 women performed 1 cycle, 69 women 2 cycles, 39 women 3 cycles, 20 women 4 cycles, 11 women 5 cycles and 13 women 6 cycles.

Follicle size was measured by calculating the diameter of the follicle on day -5 to day 0 (day -2: day of ovulation trigger; day 0: day of oocyte retrieval). E2 concentration was analysed on day -5 to day -2.

1,012 follicle size measurements and 583 E2 measurements were performed. Ovulation was triggered with 5,000 IE hCG 36 h before aspiration if the follicle size was 14–22 mm.

Women without an embryo transfer, with endometriosis ≥ rASRM II° (revised American Society of Reproductive Medicine classification of endometriosis of the American Society for Reproductive Medicine) (as diagnosed by laparoscopy or clinical and ultrasound analysis), with fibroids as diagnosed by ultrasound or with other uterine pathology (e.g., uterine polyps, adhesions) and with sperm collection by testicular sperm extraction (TESE) were excluded.

### IVF Treatments

NC-IVF cycles were monitored using transvaginal ultrasound measurements of follicular diameter and serum level of E2 and LH. When the follicle diameter reached 14–22 mm or when the E2 concentration was expected to be ≥700–800 pmol/l, 5,000 IU of hCG (Choriomon^®^, IBSA Institut Biochimique SA, Lugano, Switzerland) was administrated without LH suppression or ovarian stimulation and patients were scheduled 36 h later for oocyte retrieval (average number of days between the last consultation and HCG administration: 2.4 days (SD 0.6)). Oocyte retrieval was performed without anaesthesia and without analgesia using 19-G single lumen needles, and follicles were flushed five times (19). Embryos were transferred 2 or 3 days after aspiration. Luteal phase support was administered using vaginal micronized progesterone (Utrogestan^®^, Vifor Pharma SA, Villars-sur-Glâne, Switzerland) in the event of a short luteal phase.

Fertilization was performed by IVF or ICSI depending on the sperm quality. Clinical (defined as ultrasound detection of amniotic sac) pregnancy rates and live birth rate were analysed per transferred embryo.

### Statistical Analysis

Follicle sizes and E2 concentrations were calculated for each cycle time point. Mean differences were shown, and comparisons between the different time points were computed through a linear mixed-effect model. The overall p-values were computed by testing the equality of all the coefficients from the model with categorical time points by day. The mean increases per day were computed considering the measurement available minus the previous measurement available divided by the number of days between these two measurements.

Associations of outcome parameters for follicle sizes were derived from a multivariate mixed-effect logistic model including follicle size, oestrogen level and their interaction against different clinical variables (oocyte, mature oocyte, clinical pregnancy and birth).

Outcome parameters were adjusted for female age, cause of infertility and number of previous embryo transfers without pregnancy.

Coefficients from linear and mixed-effect linear regressions of association between E2 and LH concentrations 10 to <20 IU/l and additional coefficients for quantile mixed-effect linear regression for percentiles 25, 50 and 75 were calculated.

All analyses were performed using Stata 16 (Stata Corporation, College Station, TX).

### Ethics

Informed written consent was obtained prior to treatment, and the study was approved by the cantonal ethical committee Bern, Switzerland (KEK 2020-00634).

## Results

290 women undergoing 606 NC-IVF cycles were identified. The basic characteristics of these cycles are shown in **Table 1**. The mean female age when the cycles were performed was 35.0 ± 4 years. The overall infertility factors were male factors (48%), female factors (16%), female and male factors (14%) and idiopathic infertility (22%).

**Table 1:** Cycle characteristics (n = 606).

**Age at aspiration, years**	
Mean (SD)	35 (4.0)
Median [IQR]	36 [34, 39]
Range	22–42
**Causes of infertility, n (%)**	
Male factor	289 (48%)
Female factor	99 (16%)
Male and female	83 (14%)
Idiopathic	135 (22%)
**Number of previous embryo transfers without pregnancy, n (%)**	
0–1	457 (75%)
>2–3	107 (18%)
>4–6	42 (7%)
**AMH (ng/mL)**	
Mean (SD)	2.4 (2.4)
Median [IQR]	1.6 [0.7, 3.6]

**Figure 1:**
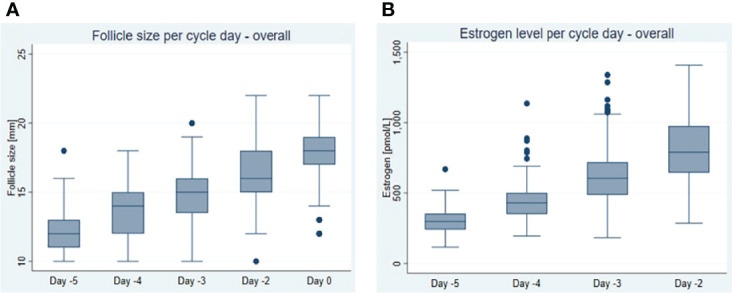
Dynamics of **(A)** follicular growth expressed as follicle size per day and **(B)** oestrogen (E2) increase expressed as oestrogen concentration per day in natural cycles within the last 5 days before follicle aspiration (day 0 = day of oocyte retrieval). Box and and whisker plot shows median, interquartile range, maximum, minimum and outliers.

Follicle diameter related to day –5 to day 0 and E2 concentrations related to day –5 to -2 are shown in **Figure 1**. On average, the follicle diameter increased at 1.04 ± 0.03 mm and E2 concentration at 167 ± 11 pmol/l per day (**Table 2**).

**Table 2:** Follicle sizes and E2 and LH concentrations on day -5 to day -2 (day -2 = day of trigger) and average increases per day.

	Day -5	Day -4	Day -3	Day -2	Day 0	Increase per day ± SD	p-value
**Follicle size, mm, mean (SD)**	12.4 (1.5)	13.5 (1.8)	14.7 (1.9)	16.5 (2.1)	17.9 (2.1)	1.04 ± 0.03	<0.001
**Follicle size, mm, median (IQ range)**	12 (11;13)	14 (12;15)	15 (14;16)	16 (15;18)	18 (17;19)	–	–
**Follicle size, observations, n**	84	189	164	146	446		
**E2, pmol/L, mean (SD)**	306 (90)	440 (133)	627 (198)	816 (219)	–	167 ± 11 (ng/ml: 46 ± 21)	<0.001
**E2, pmol/l, median (IQ-range)**	298 (241; 353)	430 (351; 501)	605 (487; 719)	790 (645; 976)	–	–	–
**E2, observations, n**	58	161	155	142	–		
**LH, IU/L, mean (SD)**	6.9 (2.8)	6.7 (2.5)	8.2 (3.0)	11.1 (3.7)	–	1.08 ± 0.30	<0.001
**LH, IU/L, median (IQ-range)**	6.4 (5.4;8.1)	6.6 (5.1;8.0)	7.8 (6.0; 9.6)	11.0 (8.6; 13.6)	–		
**LH, observations, n**	58	161	155	142	–		

Follicle size was only associated with number of mature (metaphase II) oocytes per follicle aspiration (aOR 1.27, 95% CI: 1.01–1.60) (**Table 3**). However, the E2 concentration was also associated with number of oocytes (aOR 1.91, 95% CI: 1.03–3.56). Furthermore, the association of E2 concentration with mature oocytes was more distinct (aOR 2.01, 95% CI: 1.17–3.45) than follicle size. The odds ratios for clinical pregnancy rates and live births were >1, but not significant for both parameters. Follicle size and E2 concentration interacted statistically for number of oocytes and number of mature oocytes (**Table 3**).

**Table 3:** Association of outcome parameters with follicle size and E2 concentration and interaction of both.

Outcome parameters	Follicle size, per mm, OR (95% CI)	p-value	E2 concentration, per 100pmol/l, OR (95% CI)	p-value	Interaction of follicle size and E2, OR (95% CI)	p-value
**Number of oocytes**	1.10 (0.85;1.42)	0.476	1.91 (1.03;3.56)	0.040	0.97 (0.93;1.00)	0.074
**Number of mature (MII) oocytes**	1.27 (1.01;1.60)	0.039	2.01 (1.17;3.45)	0.011	0.96 (0.93;0.99)	0.017
**Number of clinical pregnancies**	1.17 (0.78;1.75)	0.457	1.03 (0.41;2.59)	0.948	1.00 (0.94;1.06)	0.924
**Number of live births**	1.19 (0.75;1.88)	0.455	1.18 (0.43;3.24)	0.753	0.99 (0.93;1.06)	0.752

**Figure 2:**
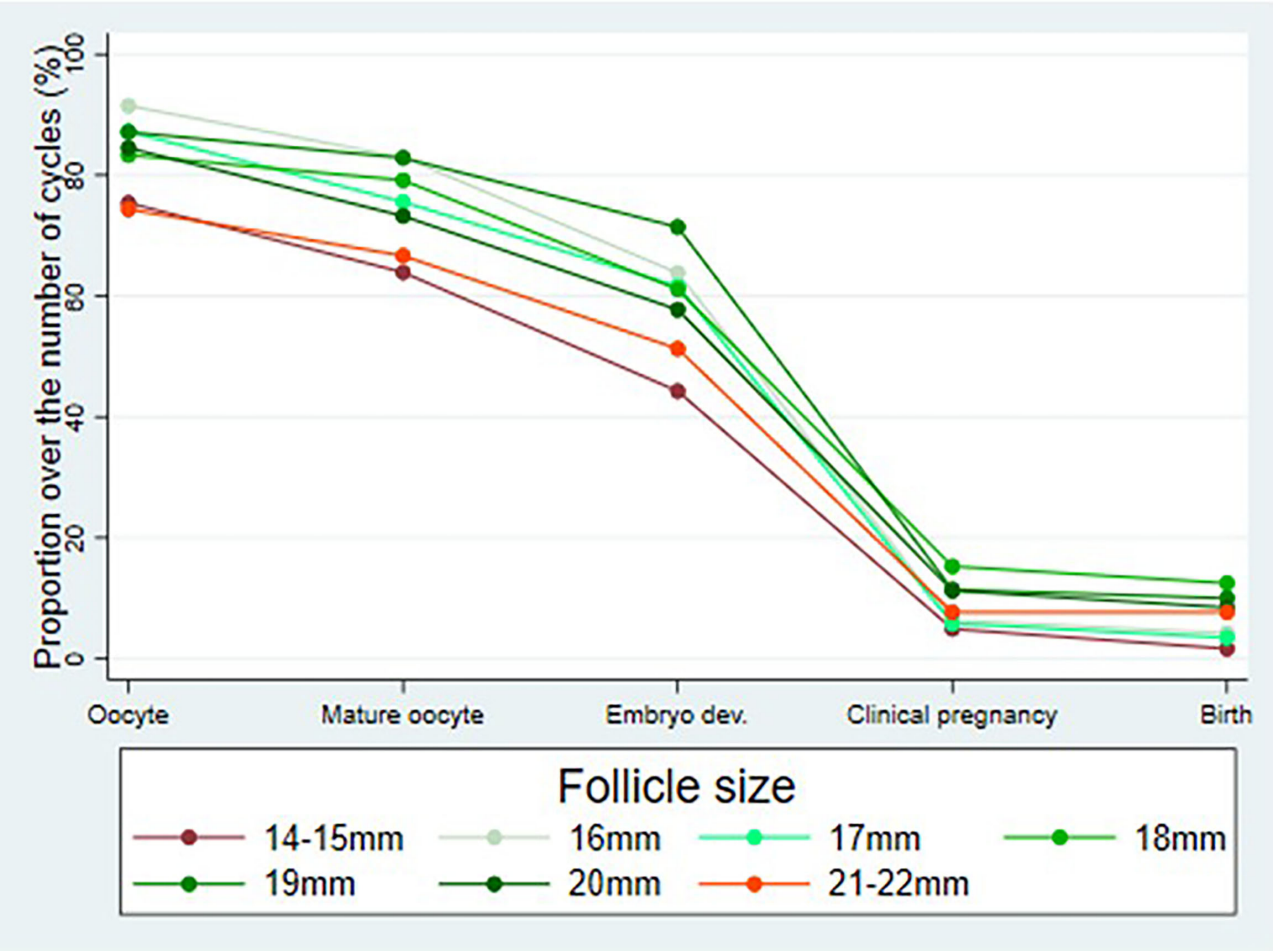
Outcome parameters (percentage of cycles with aspirated oocytes, mature oocytes (metaphase II), embryos on day 2 after follicle aspiration as well as clinical pregnancies and live births in relation to follicle size (day 0) per follicle aspiration.

**Figure 2** shows the outcome parameters in relation to follicle size per aspiration. In follicles with a diameter of 14–15 and 21–22 mm, the oocyte collection rate was lower compared to follicles 16–20 mm, leading to lower rates of mature oocytes and embryos. However, these differences were not significant.

**Table 4** describes the outcome parameters per individual parameter as mentioned in the top line of the table. The numbers do not allow definite conclusions to be drawn. However, the live birth rate per follicle aspiration cycle was (non-significantly) higher in cycles with follicles sizes at the time of oocyte retrieval of 18–22 mm (7.7%–12.5%) versus in cycles with follicles sizes of 14–17 mm (1.6%–4.3%).

**Table 4:** Association of outcome parameters with follicle size.

Follicle diameter (day of oocyte retrieval)	Number of analysed cycles	Mean (SD); median age [IQR] of the patients in years	Oocyte collection rate per aspiration (95% CI)	Oocyte MII rate per collected oocyte (95% CI)	Embryo development rate per MII oocyte (95% CI)	Clinical pregnancy rate per embryo (day 2 transfer) (95% CI)	Live birth rate per embryo (day 2 transfer) (95% CI)	Live birth rate per aspiration (95% CI)
**14–15 mm**	61	37 (3); 37 [35;39]	75.4% (63.3;84.5)	84.8% (71.8;92.4)	69.2% (53.6;81.4)	12.5% (4.3;31.0)	4.2% (0.7;20.2)	1.6% (0.3;8.7)
**16 mm**	47	38 (4); 39 [37;41]	91.5% (80.1;96.6)	90.7% (78.4;96.3)	76.9% (61.7;87.4)	10.3% (3.6;26.4)	6.9% (1.9;22.0)	4.3% (1.2;14.2)
**17 mm**	86	35 (4); 36 [32;37]	87.2% (78.5;92.7)	86.7% (77.2;92.6)	80.0% (68.7;87.9)	9.6% (4.2;20.6)	5.8% 2.0;15.6)	3.5% (1.2;9.8)
**18 mm**	72	35 (4); 36 [34;38]	83.3% (73,1;90.2)	95.0% (86.3;98.3)	77.2% (64.8;86.2)	25.0% (14.6;39.4)	20.5% (11.2;34.5)	12.5% (6.7-22.1)
**19 mm**	70	35 (4); 36 [33;38]	87.1% (77.3;93.1)	95.1% (86.5;98.3)	84.5% (73.1;91.6)	16.3% (8.5;29.0)	14.3% (7.1;26.7)	10.0% (4.9;19.2)
**20 mm**	71	35 (4); 36 [33;39]	84.5% (74.3;91.1)	86.7% (75.8;93.1)	78.8% (66.0-87.8)	20.0% (10.4;34.8)	15.0% (7.1;29.1)	8.5% (3.9;17.2)
**21–22 mm**	39	36 (4); 36 [33;38]	74.4% (58.9;85.4)	89.7% (73.6;96.4)	76.9% (57.9;89.0)	15.8% (5.5;37.6)	15.8% (5.3;37.6)	7.7% (2.7;20.3)

Adjusted for age, cause of infertility and number of previous embryo transfers without pregnancy.

**Table 5** depicts the E2 concentration at which the beginning of a LH surge can be expected, based on 138 observations with LH concentrations 10 to <20 IU/l. Based on a mixed-effect model of all data, an increase in LH can be expected in 50% of cases at an E2 concentration of 911 pmol/l. Calculations per quantile revealed that it can be expected that an LH increase occurs in 25% of cases at E2 concentrations ≤637 pmol/l. In 75% of cases, the LH increase can be expected at E2 concentrations ≤1,480 pmol/l.

**Table 5:** Association of LH and E2 concentration for defined quantiles.

Quantile	Percentage of cases with expected LH surge	pmol/L	ng/mL
**25**	**25%**	≤637	≤173
**50**	**50%**	≤911	≤248
**75**	**75%**	≤1,480	≤403

Percentage of cases with the given E2 (pmol/L and ng/mL) concentration at which beginning of LH surge (LH 10- <20 U/L) can be expected to occur.

## Discussion

Our study describes the pre-ovulatory dynamics of follicle growth, E2 increase and LH surge in spontaneous cycles and suggest the best constellation for timing of hCG triggering.

The weakness of the study is its retrospective and non-randomized design requiring multiple statistical adjustments. The strength of the study is the model of NC-IVF used in our study which not only allows us to evaluate the dynamics of follicle growth and E2 increase in the late follicular phase but also to analyse the association of these data with outcome parameters such as number of retrieved oocytes. Another strength of the study is the high number of included cycles.

The dynamics of follicle growth in spontaneous cycles has already been described in previously published papers. Renaud et al., 1980 (20), evaluated 11 cycles. The average follicular growth as measured from the time of sonographic visualization was 3 mm per day. Rossavik and Gibbons, 1985 (9), evaluated 25 cycles and found a mean daily increase in follicular size of approximately 2.5 mm. Pache et al., 1990 (21), studied 7 cycles and described an average follicle growth of 1.4 to 2.2 mm (mean 1.7 mm) per day. Baerwald et al., 2009 (10), analysed 50 cycles and found an average follicular growth of 1.48 ± 0.1 mm per day.

In contrast to these previously published studies, our study included many more cycles. Furthermore, more parameters such as the means, the medians, the standard deviations and the interquartile ranges were calculated to improve the usability of the data in clinical practice.

The previous studies found an average increase in follicle size of 3.0 mm (20), 2.5 mm (9), 1.7 mm (21) and 1.48 mm per day (10). We found an increase of 1.04 ± 0.03 mm per day. The increase in follicle size in our study was therefore lower than in the previously published studies. The reason for this discrepancy might be due to differences in the ultrasound technology, as an increase in follicles size of 3.0 mm was published in 1980 (20), of 2.5 mm in 1985 (9) and of 1.7 mm in 1990 (21). Assuming that the size of the leading follicle measures around 10 mm in the mid follicular phase cycle on day 7, as described by Bakos et al., 1994 (12), and assuming that the follicle ovulates on day 14 with a diameter of around 20 mm, as described by Hackelöer et al., 1979 (22), a theoretical average increase in follicle diameter of 1.4 mm per day would be expected. Therefore, an increase in follicle diameter of >2 mm per day as described by previously published studies can be assumed to be too high, which might be due to differences in ultrasound technology 30–40 years ago.

Our study revealed a follicle growth of even less than the theoretical expected follicle growth of 1.4 mm per day. It can be hypothesized that the even lower increase in follicle size in our study might be due to the inclusion of some cycles with dysfunctional follicles and therefore reduced follicular growth.

Hackelöer et al., 1979 (22), also studied E2 concentrations in 15 spontaneous cycles. The E2 concentration was 143 ± 25 pg/ml (526 ± 92 pmol/l) on day -5 (day 0 = LH peak) and 263 ± 46 pg/ml (967 ± 169 pmol/l) on day -1, corresponding to an increase in E2 of around 30 pg/ml (110 pmol/l) per day.

Our study revealed a much higher E2 increase of 167 pmol/l per day. The discrepancy between the study by Hackelöer et al. and our study can hardly be explained, especially as Hackelöer et al. even found a faster increase in follicle growth than described in our study, which should have led to an even more pronounced increase in E2 concentration per day in their study. This discrepancy is possibly due to different E2 assay technology, as the study by Hackelöer et al. (22) was performed around 40 years ago.

Bakos et al., 1994 (12), had already analysed the relation between the concentration of E2 and the beginning of the LH surge in spontaneous cycles. They found a mean E2 concentration of 1,087 pmol/l (range 490 to 1710 pmol/l) on the day before the LH peak and therefore on the day when the LH surge was triggered by E2. Our study revealed that the LH surge was triggered approximately by a mean E2 concentration of 911 pmol/l (interquartile range of 637 to 1,480 pmol/l). Even though the approaches by Bakos et al., 1994 (12), and our study were slightly different, the mean E2 values which trigger the LH peak are quite similar. Furthermore, both studies revealed a broad and similar range of E2 concentrations at which the LH surge can be triggered.

We also studied the association of follicle size and outcome parameters. To achieve the highest accuracy, the outcome parameters were associated with follicle size at the time of oocyte retrieval. Accordingly, to use these data in clinical practice the expected follicle sizes at the time of hCG trigger can be expected to slightly smaller than stated in **Table 4**.

Previous studies had also already analysed the association of parameters such as follicle size and rate of retrieved oocytes and implantation rate, but mainly in gonadotropin-stimulated IVF treatment cycles (14, 16). Dubey et al., 1995 (14), found a fertilization rate of 57.9% in oocytes retrieved from 10–14-mm follicles, of 69.8% in oocytes from 16–20-mm follicles and of 73.9% in oocytes retrieved from 22–26-mm follicles. The fertilization rates were significantly higher in follicles ≥16 mm compared to follicles ≤14 mm. Wirtleitner et al., 2018 (16), compared follicles sized ≥24 mm versus 13–23 mm. The oocyte retrieval rate was 81.3% vs. 76.6%, the proportion of metaphase II oocytes per retrieved oocytes was 70.1% vs. 64.0% and the zygote development rate per metaphase II oocytes was 81.4% vs. 75.3%. Even though all these rates were higher in the large follicles, the differences were not significant. Other parameters such as blastocyst development and implantation rates were neither higher nor significantly different in oocytes retrieved from larger follicles.

In unstimulated NC-IVF, such associations were previously only addressed by Reljic et al., 1999 (17). Reljic et al. studied 98 NC-IVF cycles and evaluated if the E2 concentration, measured daily between days -3 and +2 (day 0 = day of hCG trigger), was associated with oocyte retrieval rate, fertilization rate and implantation rate. They could not detect any associations.

In contrast, our study did reveal a significant association of increasing follicle size with the number of retrieved metaphase oocytes and a significant association of increasing E2 concentration with the number of retrieved oocytes and the number of retrieved metaphase II oocytes. Furthermore, our study suggests that E2 concentrations are a better prognostic factor for the retrieval of mature oocytes than the size of the follicle. DiMattina et al., 2014 (23), found a lower threshold E2 level of 408 pmol/l below that no clinical pregnancy occurred.

Our study also revealed that the live birth rate per initiated cycle was (non-significantly) higher in cycles with follicle sizes at the time of oocyte retrieval of 18–22 mm versus in cycles with follicle sizes of 14–17 mm. However, these data need to be taken with care as in some patients more than one cycle was included.

These findings are in line with the abovementioned studies performed in gonadotropin-stimulated IVF treatments regarding better outcome parameters of oocytes retrieved from follicles >14–15 mm, indicating that the impact of follicle size is similar in gonadotropin and in unstimulated cycles. In addition, we found that the E2 concentration is a better prognostic factor for the outcome of the follicle aspiration. This view is supported by previous studies which had shown that follicular fluid E2 concentrations are positively associated with embryo score in natural cycles (24).

The data provided by our and by previous studies raise the question of whether they can be used in clinical practice. The data enable the clinician to estimate the dynamics of follicle growth and to estimate the best time for the hCG trigger. Therefore, these data can be used to reduce the number of consultations to monitor follicle growth and E2 increase. As shown by von Wolff et al., 2014 (25), it is possible to perform NC-IVF with only 1.2 consultations to monitor follicle growth.

In conclusion, our study provides parameters to better estimate the dynamic of follicle growth and E2 increase in spontaneous cycles and to optimize the timing of the hCG trigger. It is thereby possible to reduce the number of follicle monitoring consultations and possibly to reduce treatment-related stress and treatment costs.

## Data Availability Statement

The raw data supporting the conclusions of this article will be made available by the authors, without undue reservation.

## Ethics Statement

The studies involving human participants were reviewed and approved by the Cantonal Ethical Committee, Bern, Switzerland (KEK 2020-00634). The patients/participants provided their written informed consent to participate in this study.

## Author Contributions

IM, PS and MvW designed the study and interpreted the data. AH and IM performed the data collection and OS the statistical analysis. MvW prepared the manuscript, and all authors revised the final manuscript. All authors contributed to the article and approved the submitted version.

## Funding

The data evaluation for this study was in part supported by an independent medical grant from IBSA Institute Biochmique SA, Lugano, Switzerland. The funding body did not have any roles in design or conduct of the study or in the preparation of the manuscript.

## Conflict of Interest

The authors declare that the research was conducted in the absence of any commercial or financial relationships that could be construed as a potential conflict of interest.

## Publisher’s Note

All claims expressed in this article are solely those of the authors and do not necessarily represent those of their affiliated organizations, or those of the publisher, the editors and the reviewers. Any product that may be evaluated in this article, or claim that may be made by its manufacturer, is not guaranteed or endorsed by the publisher.
